# Parkinson’s disease case ascertainment in prospective cohort studies through combining multiple health information resources

**DOI:** 10.1371/journal.pone.0234845

**Published:** 2020-07-01

**Authors:** Marije Reedijk, Anke Huss, Robert A. Verheij, Petra H. Peeters, Roel C. H. Vermeulen

**Affiliations:** 1 University of Utrecht, Institute for Risk Assessment Sciences, Utrecht, The Netherlands; 2 Julius Center for Health Sciences and Primary Care, University Medical Center Utrecht (UMCU), Utrecht, The Netherlands; 3 Netherlands Institute for Health Services Research (NIVEL), Utrecht, The Netherlands; Universidad Miguel Hernandez de Elche, SPAIN

## Abstract

Epidemiological evidence from prospective cohort studies on risk factors of Parkinson’s disease (PD) is limited as case ascertainment is challenging due to a lack of registries and the disease course of PD. The objective of this study was to create a case ascertainment method for PD within two prospective Dutch cohorts based on multiple sources of PD information. This method was validated using clinical records from the general practitioners (GPs). Face validity of the case ascertainment was tested for three etiological factors (smoking, sex and family history of PD). In total 54825 participants were included from the cohorts AMIGO and EPIC-NL. Sources of PD information included self-reported PD, self-reported PD medication, a 9 item screening questionnaire (Tanner), electronical medical records, hospital discharge data and mortality records. Based on these sources we developed a likelihood score with 4 categories (no PD, unlikely PD, possible PD, likely PD). For the different sources of PD information and for the likelihood score we present the agreement with GP-validated cases. Risk of PD for established factors was studied by logistic regression as exact diagnose dates were not always available. Based on the algorithm, we assigned 346 participants to the likely PD category. GP validation confirmed 67% of these participants in EPIC-NL, but only 12% in AMIGO. PD was confirmed in only 3% of the participants with a possible PD classification. PD case ascertainment by mortality records (91%), EMR ICPC (82%) and self-reported information (62–69%) had the highest confirmation rates. The Tanner PD screening questionnaire had a lower agreement (18%). Risk estimates for smoking, family history and sex using all likely PD cases were comparable to the literature for EPIC-NL, but not for smoking in AMIGO. Using multiple sources of PD evidence in cohorts remains important but challenging as performance of sources varied in validity.

## 1. Introduction

Parkinson’s disease (PD) is a neurodegenerative disease affecting a considerable part of the elderly population, with a prevalence of more than 2% in the population above 65 [1,2]. The prevalence of PD will likely increase in the coming years as the population ages and there are currently no disease modifying treatments. PD has some familial forms, however in most cases the disease is idiopathic [3]. Idiopathic PD is thought to be caused by an interaction between aging, genetics, and environmental factors [4]. For example PD is more common among men than women and a lower risk is found among smokers in epidemiological studies [5,6]. Other putative PD risk factors include pesticides, alcohol or coffee consumption and diet [7–10]. Currently many epidemiological studies on PD use a case-control design, due to the gains in efficiency and statistical power, however case-control studies may suffer from reverse causality and other biases such as retrospective recall of lifestyle factors. The lower risk of such biases in prospective cohort studies could make them an important additional resource for studying PD risk factors.

This however, raises an issue as accurate case identification of PD, a relatively rare disease, is important for the validity of effect estimates in epidemiological studies, but currently in many countries no specific registry for PD is available. This makes it a challenge to identify PD cases in prospective studies. Identification is also complicated because PD has a long preclinical period before disease manifests and an unclear moment of onset [7,11]. Further, the diagnostic procedure for PD is symptom-based, its symptoms can also be caused by related conditions such as essential tremor or non-PD parkinsonisms. Screening the total cohort population by a specialist is unfeasible and therefore, PD identification in cohort studies generally rely on medical records and self-reported information [12]. This led Tanner et al [13] to develop a series of nine questions on Parkinsonian symptoms in 1990 to help identify PD cases.

The aim of the present paper is to develop a case ascertainment method to identify Parkinson’s disease within two prospective cohort studies in the Netherlands (EPIC-NL and AMIGO). PD information was gathered from multiple sources, including the Tanner screening questionnaire [13], self-reported diagnosed PD, self-reported PD medication use, electronic medical records, hospital discharge data and mortality records. The proposed PD algorithm was tested in two manners; first the detected participants were validated against data from the general practitioners (GPs) as in the Netherlands GPs have a complete medical overview of their patients. Second, the algorithm was tested by evaluating three well studied and relatively strong etiological factors: smoking, family history of PD, and sex in this study population, termed in this paper as face validity [14].

## 2. Material and methods

### 2.1 Study population

This study was conducted within the Occupational and Environmental Health Cohort Study (In Dutch: Arbeid, Milieu en Gezondheid Onderzoek, AMIGO) and the European Prospective Investigation into Cancer and Nutrition in the Netherlands (EPIC-NL) cohort [15,16]. The recruitment procedures and study designs of these prospective cohorts are described in more detail elsewhere and in S1 Table [15,16]. Briefly, participants in AMIGO were recruited via a national general practitioners (GP) network, ie. the Netherlands Institute for Health Services Research (NIVEL) Primary Care Database in 2011 and 2012. In total 14829 adults, aged 31 to 65 years, from the general population participated. Participants received a baseline questionnaire in 2011/2012 and the first follow-up questionnaire was conducted in 2015 (n = 7905, response rate = 53%) [16]. Participants from the EPIC-NL cohort were recruited between 1993–1997, either via a breast cancer screening program conducted in the city of Utrecht and neighbouring towns (women aged 49 to 70; EPIC-Prospect), or adults aged 21 to 64 from the general population of three cities, Amsterdam, Maastricht, and Doetinchem (EPIC-Morgen), which together are referred to as EPIC-NL[15]. Participants received a baseline questionnaire between 1993–1997, in which a total of 40011 participant were included [15]. Follow-up questionnaires were conducted in the years 1998–2002 (follow-up 1, n = 28022), 2002–2003 in EPIC-Prospect only (follow-up 2, n = 12004) and 2010–2011 (follow-up 3, n = 13960) (S2 and S3 Tables). Participants included in this study signed a written (EPIC-NL) or electronic (AMIGO) informed consent. Approval was obtained from the Institutional Review Board of the University Medical Center Utrecht (EPIC-Prospect, AMIGO) and the Medical Ethical Committee of TNO Nutrition and Food Research (EPIC-Morgen). AMIGO has also been approved according to the governance code of NIVEL Primary Care Database under NZR-00317.005.

### 2.2 Data sources

**Fig 1:**
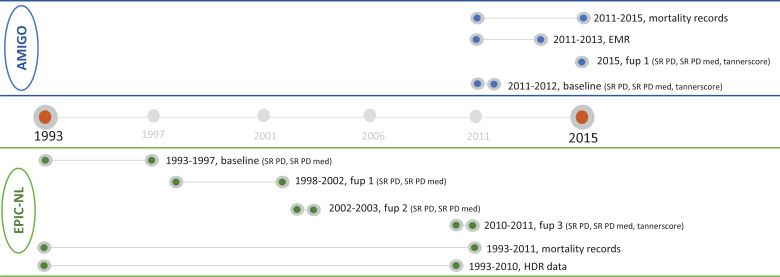
Timeline data collected within AMIGO and EPIC-NL. SR, self-reported; fup1 = follow-up 1; fup2, follow-up 2; fup3, follow-up 3; PD, Parkinson’s Disease; med, medication; EMR, electronic medical records; HDR, hospital discharge registry. Green = EPIC-NL, Blue = AMIGO.

For the development of the case ascertainment methods two types of data were used: self-reported information from the questionnaires (self-reported PD diagnosis and self-reported PD medication) and registry data (i.e., electronic medical records, hospital discharge registry, mortality records) (S4 Table). In Fig 1, the timing of the different sources of PD information is displayed. In total, the follow-up time for EPIC-NL was almost 20 years, for AMIGO it was shorter with a maximum of 5 year for mortality records.

#### 2.2.1 Self-reported information

In the questionnaires, self-reported diagnosis of PD and self-reported medication for PD were assessed. The questions were asked closed (yes/no) in the follow-up 3 questionnaire in EPIC-NL and baseline and follow-up questionnaire in AMIGO. We asked for doctor-diagnosed PD in the closed questions. The other questionnaires administered within EPIC-NL had open questions asking for diseases and medication in general.

The open medication question in the first two questionnaires of EPIC-NL were classified in Anatomical Therapeutic Chemical (ATC) codes. We searched for the ATC codes starting with: N04BA, N04BB, N04BC, N04BD and N04BX, which are specific for PD. If ATC codes were not available we identified medication by using substance names drugs and brand names of all PD drugs registered in the Netherlands as search terms. More details can be found in S5 Table.

Besides self-reported PD and self-reported PD medication a series of nine questions on Parkinsonian symptoms, such as having a smaller handwriting than before, were assessed in the baseline and follow-up questionnaire in AMIGO and the third follow-up questionnaire in EPIC-NL. This short questionnaire has been developed by Tanner et al. in 1990 and subsequently shown to be predictive for PD diagnosis in case-control studies [13,17,18]. See the full list of questions in S6 Table. From these nine questions a score is calculated by summing the number of positive items, resulting in the “Tanner score” (range 0–9). We divided the Tanner score in three categories 0–1 (unlikely), 2–4 (possible), ≥ 5 (probable), based on previous reports [18–21].

#### 2.2.2 Registry data

*Hospital discharge registry*. The Dutch hospital discharge register (HDR), is coordinated by the Dutch Hospital Association and Order of Medical Specialists [15]. The HDR is a standardized registry of hospital discharge diagnoses. It contains one mandatory principal diagnosis and up to nine additional diagnoses for every hospital discharge in the Netherlands [15]. All diagnoses in the HDR registry are classified according to the International Classification of Disease, ninth version (ICD-9) by medical administrative employees in hospitals [15]. If one of these diagnoses was PD (ICD-9-CM 332), this information was used for case ascertainment in this study. HDR diagnoses were available for EPIC-NL until 31 December 2010. As of 2011 the HDR registry changed and it is not possible to retrieve ICD coded data anymore.

*Electronic medical records*. Electronic Medical Records (EMRs) for AMIGO participants were extracted from the Netherlands Institute for Health Services Research (NIVEL) Primary Care Database [16]. In this database health outcomes are registered by the International Classification of Primary Care-1 (ICPC) and the ATC classification system registered drug prescriptions, which are combined in this study. EMR-based Parkinson’s disease is defined with ICPC-code N87. Prescriptions are defined by all ATC codes starting with N04A and N04B. EMR were available for AMIGO from 2011 to 2013.

*Mortality registry*. Causes of death of deceased were obtained via Statistics Netherlands (CBS). The cause of death register contains up to three causes of death, which are coded according to the ICD-9. If one of the reported diagnoses was PD (ICD-9-CM 332) this information was used for case ascertainment in this study. Data from the cause of death register were available until 31 December 2011 for EPIC-NL and until 31 August 2015 for AMIGO.

### 2.3 Algorithm

Based on the different sources of PD information we developed a probabilistic likelihood score to allocate participants to 4 categories: 0 = no PD, likelihood 1 = unlikely PD, likelihood 2 = possible PD, likelihood 3 = likely PD as shown in Table 1. In this score, likelihood category 1 contains Tanner scores two to four. Self-reported diagnosis, self-reported medication and a Tanner score ≥ 5 are in likelihood category 2. Likelihood 3 is a PD diagnosis in the Statistics Netherlands cause of death register, in the HDR or EMR or at least two types of information from the likelihood 2 category. Participants not matching any of these criteria are placed in likelihood category 0.

**Table 1:** Algorithm for deciding PD likelihood of participants, by available sources of PD information.

Likelihood score 1	Likelihood score 2	Likelihood score 3
Tanner score 2–4 (A1)^a^	• 1 type of evidence from:Tanner score ≥ 5 (A2)^a^ • Self-reported PD diagnosis (B)^b^ • Self-reported PD medication (C)^b^	• At least 2 types of evidence from: A2, B and C • At least: PD diagnosis death certificate (D)^c^ • At least: PD diagnosis HDR (E)^d^ • At least: PD diagnosis EMR (F)^e^

^a^ assessed in follow-up 3 questionnaire EPIC-NL, baseline and follow-up AMIGO

^b^ assessed in follow up 1 + 2 questionnaires (open questions) and follow-up 3 questionnaires (PD specific) EPIC-NL, and baseline and follow-up AMIGO

^c^ assessed from baseline to 31 December 2011 EPIC-NL, baseline to 31 August 2015 AMIGO

^d^ assessed from baseline to 31 December 2010 EPIC-NL

^e^ assessed from 2011 until 2013 in AMIGO, ATC and ICPC codes

PD, Parkinson’s Disease; EMR, electronic medical records; HDR, hospital discharge registry; ATC, Anatomical Therapeutic Chemical; ICPC, International Classification of Primary Care.

### 2.4 GP verification

The algorithm was verified using questionnaires collected between 2015 and 2017 at the GPs of participants. In the Netherlands, GPs have a complete overview of the medical status of their clients listed in their practices as they receive information back from any other health services such as specialists in hospitals. Virtually all Dutch citizens are listed with a GP as it is obligatory in the Netherlands to have a GP. The GP of the participants was contacted if participants gave informed consent and information on current GP was available. All GPs of participants in likelihood 3 were contacted. Due to restrictions on time and resources, a subsample of PD likelihood 1 and 2 were included in the validation study.

#### EPIC-NL

In EPIC-NL the GP validation was performed for all participants with likelihood 3 (n = 176) and all participants with self-reported PD diagnosis or medication data in likelihood 2 (n = 46). Additionally, a random 100 participants each from likelihood 1 and 2 were selected for GP validation (see S7 Table).

#### AMIGO

In AMIGO all participants with likelihood 3 (n = 168) were included in the GP validation study. From likelihood 2, all participants with self-reported data were selected for GP validation (n = 3) and additionally 100 randomly selected participants from likelihood 2 based on the Tanner score (S7 Table). PD is listed as a chronic disease in the EMR system and ones in the system it will remain in the system although diagnosis might be changed.

#### Procedure

In both EPIC-NL and AMIGO, general practitioners of the participants included in the validation study were contacted by mail and given a short questionnaire with eight questions. Also the GP of deceased participants were contacted as most often information in the system is still available. We largely followed the same procedure as the NeuroEPIC4PD study which has been described in detail by Gallo et al.[22]. An allowance equal to the price of a GP consult of less than 20 minutes was given to the GP per questionnaire that was returned (~9 euro). Non-responding GPs received a reminder by mail and a new copy of the questionnaire. After this reminder the non-responding GPs were contacted by telephone to answer a limited set of questions (e.g. Parkinson diagnosis and year of diagnosis).

#### Questionnaire GP validation

The most important data retrieved from the GP questionnaire was PD diagnosis and the year of diagnosis. The other questions were on the name and address of a possible new GP of the participant, name and address of the treating neurologist, PD surgery, PD medication use, PD symptoms and differential diagnosis (other forms of Parkinsonism and essential tremor).

### 2.5 Face validity

We further evaluated the performance of the algorithm by performing logistic regression with known etiological factors e.g. baseline self-reported smoking (divided into never, ever, current), sex and self-reported family history of PD.

### 2.6 Statistical analyses

From the GP validation exercise, we calculated the number and percentage of GP-confirmed cases, participants reported not to have PD and participants with a differential diagnosis (tremor by other cause or other Parkinsonism). The number and percentage GP-confirmed cases was also calculated for each separate source of PD information (i.e. self-reported PD diagnosis, self-reported PD medication, Tanner score, mortality records, HDR and EMR).

Logistic regression models were used to calculate odds ratios for known PD risk factors (first degree family history of PD, baseline smoking, sex and age). For likelihood 3, all participants with likelihood 2, 1 and 0 were taken as controls. Analyses were corrected for baseline age (continuous), education (low, medium, high), sex and cohort (in combined analyses). We also performed analyses without the variable education. We conducted the following sensitivity analyses to assess effects of possible outcome misclassification on risk estimates: 1) for PD likelihood 3, participants with likelihood 1 and 0 were taken as controls, as there may be some hidden PD cases in the second likelihood, 2) for PD likelihood 3, participants with likelihood 0 were taken as controls, as there may be some hidden PD cases in the second and first likelihood score, 3) GP validated PD cases only, with participants with likelihood 0,1 and 2 taken as controls. We also stratified analyses for cohort, age and sex. All statistical analyses were conducted using SAS, version 9.4 (SAS Institute Inc., Cary, North Carolina, USA) and R statistical software (version 3.4.3).

## 3. Results

### 3.1 Study population descriptives and PD evidences

**Fig 2:**
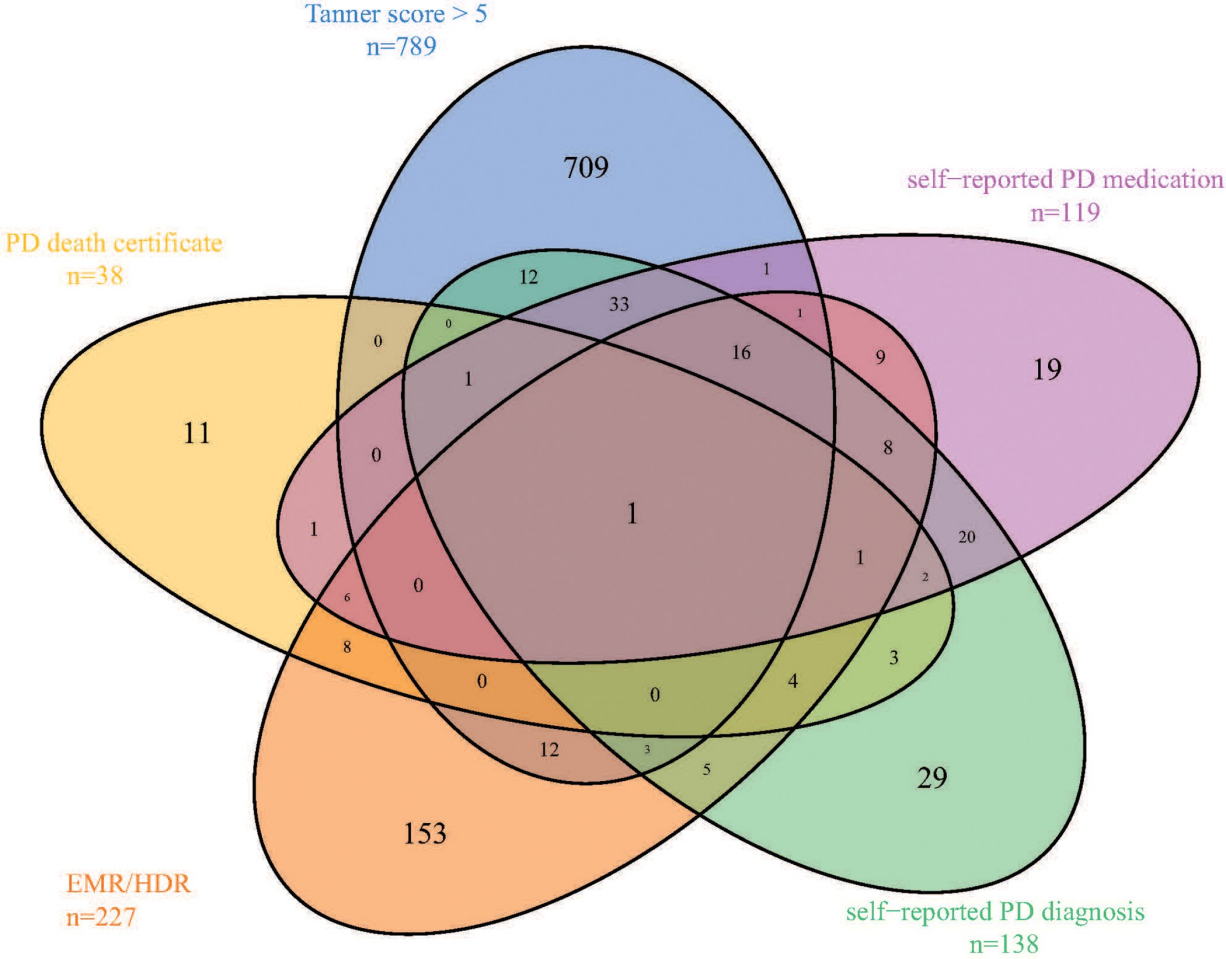
Venn diagram of different sources of PD information in the Combined cohort. PD, Parkinson’s Disease; EMR, electronic medical records; HDR, hospital discharge registry.

Baseline characteristics of the combined cohort, and for the sub cohorts AMIGO and EPIC-NL separately, are shown in Tables 2 and S8. The main differences between the two cohorts are due to differences in recruitment procedures. The higher proportion of female participants within EPIC-NL is due to the recruitment via the breast cancer screening program in EPIC-Prospect. AMIGO participants are more often higher educated and less often current smokers compared to EPIC-NL participants. The number of participants for each information source of PD is shown in Table 2, which shows that after the Tanner score, HDR and EMR are the most frequent PD information sources. Fig 2 shows the overlap between the different PD information sources for the combined cohort which indicates that participants had often only one information source of PD for the Tanner score ≥ 5 (89%) or EMR/HDR (72%). S1 and S2 Figs display the overlap for AMIGO and EPIC-NL separately which looked similar. In Table 3 the number of participants assigned to each of the four PD category is shown. In total 346 out of 54825 (0.63%) participants were assigned the highest PD likelihood.

**Table 2:** Baseline characteristics and percentage of participants with a source of PD information according to cohort (AMIGO, EPIC-NL, Combined).

	AMIGO	EPIC-NL	Combined
Total N	14829	40011	54825
Age (median, IQR)	51.0 (43.0–59.0)	51.4 (41.9–57.6)	51.4 (42.3–58.0)
Female (%)	8268 (55.8%)	29751 (74.4%)	38007 (69.3%)
Past smoker (%)	5744 (38.8%)	12440 (31.2%)	18179 (33.3%)
Current smoker (%)	2322 (15.7%)	12164 (30.5%)	14484 (26.5%)
Questionnaire data:			
Self-reported PD medication use (%)	32 (0.2%)	87 (0.2%)	119 (0.2%)
Self-reported PD doctor diagnosis (%)	26 (0.2%)	112 (0.3%)	138 (0.3%)
Tanner score 2–4 (%)	1874 (12.6%)	3068 (7.7%)	4939 (9.0%)
Tanner score ≥ 5 (%)	242 (1.6%)	547 (1.4%)	789 (1.4%)
Tanner score 2011 (mean, sd)	0.60 (1.12)	1.03 (1.48)	0.81(1.33)
Tanner score 2015 (mean, sd)	0.54 (1.05)	-	0.54 (1.05)
Registry data:			
HDR PD diagnosis (%)	-	94 (0.2%)	94 (0.2%)
EMR PD (%)	133 (0.9%)	-	133 (0.2%)
EMR PD ICPC diagnosis (%)	21 (0.1%)	-	21 (0.1%)
EMR PD ATC medication (%)	130 (0.9%)	-	130 (0.2%)
PD on death certificate (%)	1 (0.0%)	37 (0.1%)	38 (0.1%)

Participants may have multiple sources of PD information.

Age, Age at study entry; PD, Parkinson’s Disease; EMR, electronic medical records; HDR, hospital discharge registry; SD, standard deviation; IQR, Interquartile range; N, sample size; ATC, Anatomical Therapeutic Chemical; ICPC, International Classification of Primary Care.

**Table 3:** Frequency of PD likelihood scores in AMIGO, EPIC-NL and Combined cohort.

PD Likelihood	Cohort	Number (%)
**0**	AMIGO	12193 (82.2%)
	EPIC-NL	36260 (90.6%)
	Combined	48442 (88.4%)
**1**	AMIGO	2223 (15.0%)
	EPIC-NL	3039 (7.6%)
	Combined	5258 (9.6%)
**2**	AMIGO	243 (1.6%)
	EPIC-NL	536 (1.4%)
	Combined	779 (1.4%)
**3**	AMIGO	170 (1.2%)
	EPIC-NL	176 (0.4%)
	Combined	346 (0.6%)

### 3.2 GP validation of the PD likelihood score

A sample of 668 participants out of 6383 participants with a PD likelihood score of 1 or higher were selected for GP validation (10%). For 167 participants, there was no information on PD available from the GP either by a lack of information the GP had available (n = 125) or because there was no response from the GP (n = 42). Table 4 provides an overview of the number of participants that were confirmed having PD by their GP. Of the 501 participants with information on PD diagnosis, 85 were confirmed to have PD (17%). None of the likelihood 1 participants had a PD diagnosis confirmed by their GPs. In total 5 (3%) cases of likelihood 2 were confirmed having PD and 14 (7%) participants were reported to have tremor by another cause than PD or another form of Parkinsonism. 34% of the participants with a GP questionnaire returned in likelihood 3 were confirmed to have a PD diagnosis. There were large differences between AMIGO and EPIC-NL, as respectively 12% and 67% of likelihood 3 participants were confirmed by their GP. PD confirmed cases by their GP were older and had more often family with a history of PD (S9 Table) compared to non-confirmed cases.

**Table 4:** Validation of PD Likelihood scores by General Practitioner for EPIC-NL, AMIGO and Combined cohort.

**EPIC-NL**						
PD Likelihood	Participants selected for validation	Returned GP questionnaire (% of participants selected)	Information available diagnosis (% of participants selected)	PD diagnosis confirmed by GP (%)	No PD diagnosis by GP (%)	Other Parkinsonism/ tremor by other cause (%)
1	99	96 (97%)	72 (73%)	0 (0%)	72 (100%)	2 (3%)
2	141	135 (96%)	110 (78%)	5 (5%)	105 (95%)	6 (5%)
3	160	152 (95%)	93 (58%)	62 (67%)	31(33%)	8 (9%)
**AMIGO**						
PD Likelihood	Participants selected for validation	Returned GP questionnaire (% of participants selected)	Information available on PD diagnosis (% of participants selected)	PD diagnosis confirmed by GP (%)	No PD diagnosis by GP(%)	Other Parkinsonism/ tremor by other cause (%)
2	100	92 (92%)	81 (81%)	0 (0%)	81 (100%)	8 (10%)
3	168	153 (91%)	145 (86%)	18 (12%)	127 (88%)	15 (10%)
**Combined**						
PD Likelihood	Participants selected for validation	Returned GP questionnaire (% of participants selected)	Information available on PD diagnosis (% of participants selected)	PD diagnosis confirmed by GP (%)	No PD diagnosis by GP (%)	Other Parkinsonism/ tremor by other cause (%)
1	99	96 (97%)	72 (73%)	0 (0%)	72 (100%)	2 (3%)
2	241	227 (94%)	191 (79%)	5 (3%)	186 (97%)	14 (7%)
3	328	305 (93%)	238 (73%)	80 (34%)	158 (66%)	23 (10%)

PD, Parkinson’s Disease; GP, general practitioner

### 3.3 Validation of PD information sources

A PD diagnosis on a death certificate was found in 10 cases (90.9%) to correspond with a GP-confirmed diagnosis, but only 11.7% of all PD cases were identified on the basis of a death certificate (Tables 5 and S10). The agreement was also high for self-reported PD diagnosis (62.4%) and self-reported PD medication (68.5%). The agreement for EMR was 14% with large differences between EMR based on ATC medication codes (12.6%) or ICPC diagnosis code (82.4%). The mean Tanner score was higher for GP validated cases than non-confirmed cases (S10 Table). The mean age at diagnosis for validated GP cases was 59 years (range 34 to 68) in AMIGO compared to 68 years (range 48 to 85 year) in EPIC-NL (S9 Table).

**Table 5:** Verification of different sources of PD information by information retrieved from the General Practitioners for AMIGO, EPIC-NL and Combined cohort.

Information source PD	N evidence available	N positive evidence	%	95%CI	Confirmed PD status at GP		
					PD	%	95%CI
AMIGO	226				18		
PD diagnosis (SR)	226	21	9.3	5.8–13.9	15	71.4	47.8–88.7
PD medication(SR)	226	26	11.5	7.7–16.4	15	57.7	36.9–76.6
Tanner score 2011 ≥ 2	226	162	71.7	65.3–77.5	15	9.3	5.3–14.8
Tanner score 2011 ≥ 5	226	108	47.8	41.1–54.5	10	9.3	4.5–16.4
PD EMR	226	114	50.4	43.7–57.1	16	14.0	8.2–21.8
PD EMR ICPC diagnosis	226	17	7.5	4.4–11.8	14	82.4	56.6–96.2
PD EMR ATC medication	226	111	49.1	42.4–55.8	14	12.6	7.1–20.3
PD on death certificate	226	1	0.4	0.0–2.4	1	100	2.5–100
EPIC-NL	275				67		
PD diagnosis (SR)	275	72	26.2	21.1–31.8	43	59.7	47.5–71.1
PD medication(SR)	275	47	17.1	12.8–22.1	35	74.5	59.7–86.1
Tanner score 2011 ≥ 2	233	211	90.6	86.1–94.0	36	17.1	12.2–22.8
Tanner score 2011 ≥ 5	233	116	49.8	43.2–56.4	31	26.7	18.9–35.7
PD HDR	275	42	15.3	11.2–20.1	23	54.8	38.7–70.2
PD on death certificate	275	10	3.6	1.8–6.6	9	90.0	55.5–99.7
COMBINED	501				85		
PD diagnosis (SR)	501	93	18.6	15.3–22.2	58	62.4	51.7–72.2
PD medication(SR)	501	73	14.6	11.6–18.0	50	68.5	56.6–78.9
Tanner score 2011 ≥ 2	459	373	81.3	77.4–84.7	52	13.9	10.6–17.9
Tanner score 2011 ≥ 5	459	224	48.8	44.1–53.5	41	18.3	13.5–24.0
PD on death certificate	501	11	2.2	1.1–3.9	10	90.9	58.7–99.8

SR, self-reported; PD, Parkinson’s Disease; EMR, electronic medical records; HDR, hospital discharge registry; GP, general practitioner; ATC, Anatomical Therapeutic Chemical; ICPC, International Classification of Primary Care.

### 3.4 Association with known etiological factors—face validity

**Fig 3:**
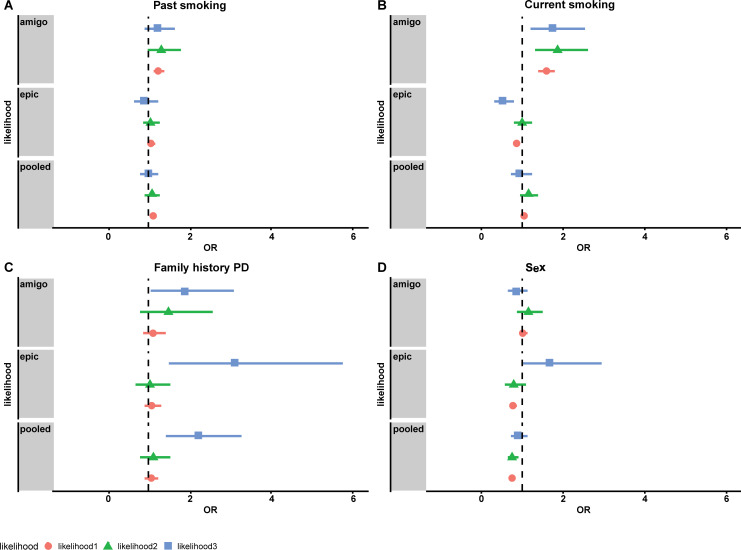
Adjusted logistic regression by PD likelihood scores for known PD risk factors past smoking (baseline)(A), current smoking (baseline)(B), 1^st^ degree family history of PD (C), and sex (D) in AMIGO, EPIC-NL and Combined cohort. Reference group for smoking were never smokers. PD, Parkinson Disease; OR, odds ratio. Likelihood 3 compared to likelihood 0. Likelihood 2 compared to likelihood 0. Likelihood 1 compared to likelihood 0. * Adjusted for age at baseline, baseline educational level, sex and cohort (combined analyses).

Characteristics for likelihood 3 compared to likelihood 0–2 are shown in S11 and S12 Tables. The results of the face validity for smoking, sex and family history for PD likelihood scores 1–3 compared to likelihood 0 are shown in Fig 3 and S13 and S14 Tables for likelihood 3. Current smoking (at baseline) as compared to never smoked showed an OR of 0.88(95% CI: 0.65–1.18) for likelihood 3 (compared to likelihood 0–2). Similarly, for past smokers, only for likelihood 3 risk estimates below unity were observed, compared to never smokers. Large differences existed between the two cohorts for past and current smoking, the estimates found in EPIC-NL were noticeably lower. First degree family history of PD increased the risk of PD for likelihood 3 with an OR of 2.29 (95% CI: 1.43–3.49) in the Combined cohort (S13 Table). PD risk of sex (male vs. female) provided an OR of 0.79 (95% CI: 0.57–1.07) for AMIGO. The cohort specific odds ratio in EPIC-NL was 1.56 (95%CI: 0.90–2.76) for male vs female.

Sensitivity analyses with likelihood 0–1 and likelihood 0 instead of likelihood 0–2 as controls for likelihood 3 showed very similar results (S14 Table). The results of the face validity analyses for smoking, sex and PD family history for the GP confirmed PD cases (n = 85) was similar to the results of likelihood 3 cases (n = 346) except for sex, although statistical power was low (S15 Table). Sensitivity analyses without EMR ATC medication in likelihood 3 for AMIGO showed that the sex association reversed to 1.65 (95%CI: 1.02–2.71) compared to 0.81(95%CI: 0.59–1.10) with EMR medication in the highest likelihood (S16 Table). Stratified analysis by sex showed higher ORs for PD family history for females (S17 Table). Stratification by age showed differences for smoking and PD family history but these findings were not consistent in the two cohorts (S17 Table).

## 4. Discussion

In this paper, we combined multiple sources of health information regarding Parkinson’s disease to assign participants a probabilistic likelihood score for PD. We validated this algorithm against information retrieved from general practitioners and computed risk estimates for three well-established risk factors (smoking, sex and family history) of PD to judge whether they were in line with expectation.

### 4.1 GP validation

In our study 0.63% of the participants were assigned to the highest likelihood score which is lower than the 1.4% expected prevalence in the general population above 55 [23,24]. If we combine self-reported PD and self-reported PD medication with likelihood 3 (n = 394, 0.72%) the prevalence in our cohort slightly increases. A possible explanation for the lower prevalence is that in EPIC-NL a healthy volunteer effect has been observed [25] and our study population (especially in the AMIGO cohort) is younger. Besides there is a possibility of unidentified PD cases in the lower likelihood scores.

In total 85 participants were diagnosed with PD by their general practitioner. We were unable to verify the PD status of 167 participants invited for GP validation. From likelihood 2 a modest 3% of the participants were confirmed to have PD. The highest likelihood category had a verification rate of 34%. Verification rates for AMIGO and EPIC-NL differed considerably as the agreement for EPIC-NL participants was 67% and that of AMIGO was 12% for likelihood 3. In addition to comparing the PD likelihood score to GP clinical records we also compared the different sources of PD information against GP information. Self-reported PD medication had a high agreement as 69% were confirmed by their GP. Mortality records from the Statistics Netherlands cause of death registry only found 10 of the 85 GP confirmed cases (12%) but the ones identified had a high likelihood of having PD (91%). Two other registries used in this study were the EMR and the HDR in AMIGO and EPIC-NL respectively. A positive PD diagnosis in the EMR had a much lower agreement with GP validated cases than the HDR (14% compared to 55%). The EMR system found 16 out of the 18 PD validated cases in AMIGO, but also 98 participants were identified as having PD by the EMR system but not by the GP. This could be caused by the fact that if a GP suspects PD in a patient, it is noted in the EMR system where it remains as PD is regarded as a chronic disease. Alternatively, PD medication is sometimes used as a diagnostic tool for PD which may have led to a higher amount of false positives in AMIGO. This is supported by the lower agreement found for ATC medication codes in comparison to ICPC diagnosis code which showed a high agreement with the GP records (82%).

PD likelihood scores 1 and 2 were largely assigned because of the reporting of PD symptoms via the screening questionnaire of Tanner. There were only 5 participants with likelihood score 2 that were confirmed to have PD by their GP, so Tanner by itself was not predictive for PD in a prospective setting. It is possible that after a longer period of follow-up the verification rates will increase since the screenings questionnaire was administered only a couple of years (±4) before the end of follow-up in both EPIC-NL and AMIGO.

### 4.2 Face validity of likelihood algorithm

The effect estimates of three well-known risk factors (smoking, sex, and family history of PD) were similar to what we expected based on the literature for the highest likelihood in EPIC-NL. However, estimates were attenuated towards the null for smoking and reversed for sex in AMIGO. A possible explanation for the attenuated finding for smoking in AMIGO is that ever or currently smoking participants may have had more medical visits due to smoking-related medical conditions, and this provides more opportunity for PD to be identified. The PD risk of first degree family history was OR 2.29 (95% CI: 1.43–3.49) for likelihood 3 in the Combined cohort and was slightly smaller than the odds ratio found in the meta-analysis of Noyce et al. [14]. Males had comparable PD risk to women in our study, while the previous literature showed higher risk of PD for males. However, the estimate of EPIC-NL (OR: 1.56, 95% CI: 0.90–2.76) was comparable with the OR found in a systemic review on sex and PD from 2004 [26]. A reversed effect estimate was found for sex in AMIGO. However, if we eliminated cases identified based on EMR medication the effect estimate for sex was in the expected direction and of similar magnitude (S17 Table). In our study, different effects estimates were found for the two different cohorts. The effect estimates found in EPIC-NL for the face validity, which were in the expected direction, and the high agreement of likelihood 3 by the GP in EPIC-NL indicate that the algorithm worked well in identifying PD cases. However the effect estimates of AMIGO were attenuated or even reversed for sex of what we expected and likelihood 3 had a much lower agreement with GP validated cases (12%). This means that the proposed algorithm was less capable in identifying PD cases in AMIGO. A possible explanation for this is the performance of the EMR based on ATC medication codes. 133 participants had a positive result in the EMR registry based on ATC codes or ICPC diagnosis, which was an important source to end up in the highest likelihood in AMIGO. The EMR registry on ATC medication codes had an agreement of only 13%. Therefore, ATC medication codes should be reconsidered in future use of the PD algorithm for AMIGO. The risk of PD increases with age, which was also seen in our study. Baseline age was comparable in both cohorts but as EPIC-NL had 15 years longer follow-up time, the age of EPIC-NL participants at the end of follow-up was higher. This is not reflected in the tables (results section) because only age at baseline is reported.

The past couple of years more effort is conducted on ascertainment of PD in population-based cohort studies [12,22,27,28]. These studies were also conducted originally for other health outcomes, such as the Cardiovascular Health Study [12,28], and Framingham Heart Study [27]. They indicate that using multiple sources of PD information is important but also that self-reported data remains important in cohort studies as medical registry data have limitations and on average had lower agreements, similar as our study[27,28].

### 4.3 Strengths and limitation

For PD ascertainment we used multiple sources of PD information retrieved at multiple time points during on-going prospective studies which was a strength as disease misclassification can be reduced by multiple sources [28–30]. Using only one type of evidence might cause a proportion of the cases not to be identified [2,5]. Another strength was the long follow-up period of more than 20 year in EPIC-NL. A long follow-up period is important because of the long preclinical period of PD [31]. Another strength was the large sample size, making this one of the largest cohort studies on Parkinson’s disease in the Netherlands. Our study was limited by the fact that we only validated a part of our study population and not all GPs responded to our questionnaire. We only selected 668 participants out of the total study population of 54825 for GP validation and therefore we were not able to calculate population prevalence, sensitivity, specificity, positive and negative predictive values or c-statistics. Second, by validating only this subsample there might be unidentified cases of PD that were not included in the sub-sample. However, for a relatively rare disease like PD, unidentified cases do not have much influence on the risk estimates in a cohort study as the effect of these participants is diluted because of a large surplus of true controls [32]. Nevertheless, the PD cases that are identified must be representative of all cases as to not bias risk estimates. Another weakness of the case ascertainment method regarding disease misclassification is the possibility of ascertaining cases that are not truly PD cases. Unfortunately, verification of PD status by a neurologist, the golden standard in PD research, was not possible in this study setup. We verified the algorithm against information retrieved from the GPs, which is after neurological examination by a physician, the best way of validation in the Netherlands because GPs are regarded to have a complete overview of the medical status of patients including information from the treating neurologist. However, there could be delays and omissions in the GP system.

### 4.4 Conclusion

We applied a case ascertainment strategy for PD in two prospective cohort studies, which generated a probabilistic likelihood score to classify participants into four categories. The highest likelihood performed reasonably well when comparing the obtained effect estimates of known PD risk factors with the literature, particular in EPIC-NL. The case ascertainment algorithm worked well to identify participants with PD in EPIC-NL as likelihood 3 had a GP confirmation rate of 67%. The AMIGO algorithm can be improved by not incorporating recorded PD medication use which generated most false positives. Overall, self-reported information and PD evidence in the mortality registry performed well with high agreements (62%-91%). Other sources of PD information gave varying results and performance is dependent on the source and underlying cohort demographics.

## Supporting information

S1 Table Cohort characteristics of EPIC-NL (EPIC-MORGEN, EPIC-PROSPECT) and AMIGO. (DOCX)

S2 Table Timing of questionnaires of EPIC-PROSPECT, EPIC-MORGEN, AMIGO. (DOCX)

S3 Table Questionnaire response rates in EPIC-NL (EPIC-PROSPECT, EPIC-MORGEN) and AMIGO. (DOCX)

S4 Table Different sources of PD information in EPIC-NL and AMIGO. (DOCX)

S5 Table Substance names and brand names used as search terms in EPIC-NL. (DOCX)

S6 Table Questions of the Tanner Questionnaire in AMIGO and EPIC-NL. (DOCX)

S7 Table Frequency of participants per likelihood score selected for GP follow-up in EPIC-NL and AMIGO. (DOCX)

S8 Table Baseline characteristics of AMIGO, EPIC-NL and Combined cohort. (DOCX)

S9 Table Baseline characteristics PD versus no PD validated by GP for Combined cohort. (DOCX)

S10 Table Agreement different sources of PD information and PD status, among cases validated by the GP in AMIGO, EPIC-NL and Combined cohort. (DOCX)

S11 Table Baseline characteristics likelihood 3 versus likelihood 0–2 in the Combined cohort. (DOCX)

S12 Table Baseline characteristics of likelihood 3 versus likelihood 0–2 for EPIC-NL and AMIGO. (DOCX)

S13 Table Crude and adjusted logistic regression analysis of likelihood 3 compared to likelihood 0–2 for the risk factors smoking (baseline), 1^st^ degree family history of PD, and sex in AMIGO, EPIC-NL and Combined cohort. (DOCX)

S14 Table Crude and adjusted logistic regression analysis of likelihood 3 compared to likelihood 0–1 and likelihood 3 compared to likelihood 0 for the risk factors smoking (baseline), 1^st^ degree family history of PD, and sex in AMIGO, EPIC-NL and Combined cohort. (DOCX)

S15 Table Crude and adjusted logistic regression analysis of confirmed cases by GP for the risk factors smoking (baseline), 1^st^ degree family history of PD, and sex in Combined cohort. (DOCX)

S16 Table Likelihood 3 with and without medication diagnosis in the electronic medical registry compared to likelihood 0–2 for the risk factors smoking (baseline), 1^st^ degree family history of PD, and sex within AMIGO. (DOCX)

S17 Table Logistic regression analysis of likelihood 3 compared to likelihood 0 for the risk factors smoking (baseline), 1^st^ degree family history of PD in AMIGO, EPIC-NL and Combined cohort stratified by sex and age. (DOCX)

S1 Fig Venn diagram of different sources of PD information in AMIGO. (EPS)

S2 Fig Venn diagram of different sources of PD information in EPIC-NL. (EPS)
