# Supplementary material for: Parkinson’s disease case ascertainment in prospective cohort studies through combining multiple health information resources
Source: PLoS One. 2020 Jul 1;15(7):e0234845. doi: 10.1371/journal.pone.0234845 (PMC7329061; doi:10.1371/journal.pone.0234845)
Supplement: S1 Table — (DOCX) [file pone.0234845.s001.docx]

**Table S1.** Cohort characteristics of EPIC-NL (EPIC-MORGEN, EPIC-PROSPECT) and AMIGO.

| Cohort characteristics | EPIC-MORGEN | EPIC-PROSPECT | AMIGO |
| --- | --- | --- | --- |
| Type of participants and source population | General population sample of men and women age 20-59 at recruitment | Women aged 49–70 at recruitment, who received a call-up for the Dutch breast cancer screening | General population sample of men and women age 31-65 at recruitment |
| Place of recruitment | Cities of Amsterdam, Doetinchem and Maastricht | City of Utrecht and its vicinity | Whole of the Netherlands |
| Participation rate at baseline | 44.6% | 34.5% | 16% |
| Number of participants | 22,654 | 17,357 | 14,829 |
| Time of recruitment | 1993-1997 | 1993-1997 | 2011-2012 |
| Procedure of recruitment | Sample of men and women from the general population and included those who agreed when asked for participation. | Women selected (by age) for breast cancer screening were asked for participation by filling in two questionnaires. Those that returned the questionnaires were asked to participate. | Men and women recruited a general practitioners network, with a maximum of one person per household. |
