# Supplementary material for: Parkinson’s disease case ascertainment in prospective cohort studies through combining multiple health information resources
Source: PLoS One. 2020 Jul 1;15(7):e0234845. doi: 10.1371/journal.pone.0234845 (PMC7329061; doi:10.1371/journal.pone.0234845)
Supplement: S2 Table — (DOCX) [file pone.0234845.s002.docx]

**Table S2.** Timing of questionnaires of EPIC-PROSPECT, EPIC-MORGEN, AMIGO.

| Questionnaire | EPIC-MORGEN | EPIC-PROSPECT | AMIGO |
| --- | --- | --- | --- |
| Baseline | 1993-1997 | 1993-1997 | 2011-2012 |
| Follow-up 1 | 1998-2002 | 1998-2000 | 2015 |
| Follow-up 2 | 2003-2007 (Doetinchem only) | 2002-2003 |  |
| Follow-up 3 | 2010-2011 (Amsterdam + Maastricht) | 2011 |  |
