# Supplementary material for: Parkinson’s disease case ascertainment in prospective cohort studies through combining multiple health information resources
Source: PLoS One. 2020 Jul 1;15(7):e0234845. doi: 10.1371/journal.pone.0234845 (PMC7329061; doi:10.1371/journal.pone.0234845)
Supplement: S3 Table — (DOCX) [file pone.0234845.s003.docx]

**Table S3.** Questionnaire response rates in EPIC-NL (EPIC-PROSPECT, EPIC-MORGEN) and AMIGO.

|  | AMIGO | | | | EPIC-PROSPECT | |  | EPIC-MORGEN | |  | EPIC-NL | |
| --- | --- | --- | --- | --- | --- | --- | --- | --- | --- | --- | --- | --- |
|  | *No. of participants* | | | *% of baseline* | *No. of participants* | *% of baseline* |  | *No. of participants* | *% of baseline* |  | *No. of participants* | *% of baseline* |
| ***Baseline*** | 14829 | | |  | 17357 | - |  | 22655 | - |  | 40011 | - |
|  |  |  |  | |  |  |  |  |  |  |  |  |
| ***FU1*** | 7905 | | | 53% | 13201 | 76.1 % |  | 14821 | 65.4% |  | 28022 | 70.0% |
| ***FU2*** |  | | |  | 12004 | 69.0% |  | - | - |  | - | - |
| ***FU3*** |  | | |  | 7784 | 44.8% |  | 6176 | 27.3% |  | 13960 | 34.9% |
