# Supplementary material for: Parkinson’s disease case ascertainment in prospective cohort studies through combining multiple health information resources
Source: PLoS One. 2020 Jul 1;15(7):e0234845. doi: 10.1371/journal.pone.0234845 (PMC7329061; doi:10.1371/journal.pone.0234845)
Supplement: S4 Table — (DOCX) [file pone.0234845.s004.docx]

**Table S4.** Different sources of PD information in EPIC-NL and AMIGO.

| Evidence | EPIC-NL | AMIGO |
| --- | --- | --- |
| Self-reported PD diagnosis | Yes: baseline, follow-ups | Yes: baseline, follow-up |
| Self-reported PD medication | Yes: baseline, follow-ups | Yes: Baseline, follow-up |
| Tanner questionnaire | Yes: follow-up 3 | Yes: Baseline, follow-up |
| HDR | Yes: until 31 December 2010 | No |
| EMR | No | Yes: from 2011 till 2013 |
| Mortality | Yes: until 31 December 2011 | Yes: until 31 December 2015 |

PD, Parkinson’s Disease; EMR, electronic medical records; HDR, hospital discharge registry.
