# Supplementary material for: Parkinson’s disease case ascertainment in prospective cohort studies through combining multiple health information resources
Source: PLoS One. 2020 Jul 1;15(7):e0234845. doi: 10.1371/journal.pone.0234845 (PMC7329061; doi:10.1371/journal.pone.0234845)
Supplement: S5 Table — (DOCX) [file pone.0234845.s005.docx]

### **Table S5.** Substance names and brand names used as search terms in EPIC-NL.

ADARTREL

AKINETON

ALMIRIDE

AMANTADINE

APOBENZTROP

APOGO

APOKYN

APOMORFINE HYDROCHLORIDE

APOTRIHEX

APRICOLIN

ARNALEVOCAP

ARTANE

ATURBAN

AZILECT

BENSERAZIDE

BENZATROPINE

BENZHEXOL

BENZTROP

BIPERIDEEN

BORNAPRINE

BROMOCRIPTINE

BUDIPINE

CABASER

CABERGOLINE

CABERLIN

CARBIDOPA

CLARIUM

CO-BENELDOPA

CO-CARELDOPA

COGENTIN

COMTAN

COMTESS

CORBILTA

CRIPAR

CYCLOSET

DACEPTON

DAQUIRAN

DEKINET

DEPRENALINE

DEPRENIL

DEPRENYL

DEXETIMIDE

DIHYDROERGOCRYPTINE

DISIPAL

DISIPALETTE

DOPERGIN

DOSTINEX

DOSTINEX

DUODOPA

ELDEPRIL

ELDEPRYL

ENCAPIA

ENTACAPON

ENTACAPONE

ETANAUTINE

GLEPARK

IXENSE

JUMEX

KEMADRIN

L-DOPA

LEGANTO

LEPTICUR

LEVODOPA

LEVODOPUM

LISURIDE

MADOPAR

MELEVODOPA

METIXENE

MIRAPEX

MIRAPEXIN

MODOPAR

NAUTAMINE

NEUPRO

OPRYMEA

ORFENADRINE

ORPHENADRINE

PACITANE

PARA LEST

PARKIN

PARLODEL

PARSIDAN

PARSIDOL

PERGOLIDE

PERGOLIDEMESILAAT

PERMAX

PHENGLUTARIMIDE

PIRIBEDIL

PLAMIPEXOL

PRAMIPEXOLE

PRAMITHON

PROCLACAM

PROCYCLIDINE

PROFENAMINE

PROLOPA

PRONORAN

RASAGILINE

REPREVE

REQUIP

REVANIL

RONIROL

ROPINIROL

ROTIGOTINE

SELEGILINE

SINEMET

SIFROL

SORMODREN

SPONTANE

STALEVO

SYMMETREL

TASMAR

TOLCAPON

TRASTAL

TREMARIL

TREMBLEX

TRIHEX

TRIHEXYFENIDYL

TRIVASTAL

TRIVASTAN

TROPATEPINE

UPRIMA

ZELAPAR
