# Supplementary material for: Parkinson’s disease case ascertainment in prospective cohort studies through combining multiple health information resources
Source: PLoS One. 2020 Jul 1;15(7):e0234845. doi: 10.1371/journal.pone.0234845 (PMC7329061; doi:10.1371/journal.pone.0234845)
Supplement: S6 Table — (DOCX) [file pone.0234845.s006.docx]

**Table S6**. Questions of the Tanner Questionnaire in AMIGO and EPIC-NL.

| No. | Question ^a^ |
| --- | --- |
| 1. | Do you have trouble arising from a chair? |
| 2. | Is your handwriting smaller than it once was? |
| 3. | Do people tell you that your voice is softer than it once was? |
| 4. | Is your balance poor? |
| 5. | Do your feet ever seem to get stuck to the floor? |
| 6. | Do people tell you that your face seems less expressive than it once did? |
| 7. | Do your arms and legs shake? |
| 8. | Do you have trouble buttoning buttons? |
| 9. | Do you shuffle your feet and/or take tiny steps when you walk |

^a^ A Dutch translation of the questionnaire was used.
