# Supplementary material for: Parkinson’s disease case ascertainment in prospective cohort studies through combining multiple health information resources
Source: PLoS One. 2020 Jul 1;15(7):e0234845. doi: 10.1371/journal.pone.0234845 (PMC7329061; doi:10.1371/journal.pone.0234845)
Supplement: S7 Table — (DOCX) [file pone.0234845.s007.docx]

**Table S7**. Frequency of participants per likelihood score selected for GP follow-up in EPIC-NL and AMIGO.

|  | EPIC-NL | | AMIGO |  |
| --- | --- | --- | --- | --- |
| Likelihood | Frequency | Selected for GP follow-up | Frequency | Selected for GP follow-up |
| 0 | 36260 | 0 | 12193 | 0 |
| 1 | 3039 | 100 (random) | 2223 | 0 |
| 2: TS | 490 | 100 (random) | 240 | 100 (random) |
| 2: other* | 46 | 46 | 3 | 3 |
| 3 | 176 | 176 | 170 | 170 |

* self-reported Parkinson disease or self-reported medication.
TS, Tanner questionnaire Score; GP, general practitioner
