# Supplementary material for: Parkinson’s disease case ascertainment in prospective cohort studies through combining multiple health information resources
Source: PLoS One. 2020 Jul 1;15(7):e0234845. doi: 10.1371/journal.pone.0234845 (PMC7329061; doi:10.1371/journal.pone.0234845)
Supplement: S8 Table — (DOCX) [file pone.0234845.s008.docx]

**Table S8**. Baseline characteristics of AMIGO, EPIC-NL and Combined Cohort.

|  | EPIC-NL | AMIGO | COMBINED |
| --- | --- | --- | --- |
| *Number of participants (%)* | 40011 | 14829 | 54825 |
| *Age at baseline* |  |  |  |
| Mean (SD) | 49.21(11.90) | 50.65(9.37) | 49.60(11.29) |
| *Sex (%)* |  |  |  |
| Male | 10260(25.6%) | 6561(44.2%) | 16818(30.7%) |
| Female | 29751(74.4%) | 8268(55.8%) | 38007(69.3%) |
| *Education (%)* |  |  |  |
| Low | 24198(61.0%) | 4537(30.6%) | 28731 (52.7%) |
| Medium | 7407(18.7%) | 4627(31.2%) | 12033(22.1%) |
| High | 8095(20.4%) | 5656(38.2%) | 13741(25.2%) |
| Missing | 311 | 9 | 320 |
| *Smoking status at baseline (%)* |  |  |  |
| Never smoker | 15243 (38.3%) | 6740(45.5%) | 21975(40.2%) |
| Past smoker | 12440(31.2%) | 5744(38.8%) | 18179(33.3%) |
| Current smoker | 12164(30.5%) | 2322(15.7%) | 14484(26.5%) |
| Missing | 164 | 23 | 187 |
| Family history PD 1^st^ degree (%)* |  |  |  |
| Yes | 638(4.6%) | 496(3.3%) | 1133(3.9%) |

*Only available for follow-up 3 in EPIC-NL, % calculated based on these participants.
PD, Parkinson’s Disease; SD, standard deviation
