# Supplementary material for: Parkinson’s disease case ascertainment in prospective cohort studies through combining multiple health information resources
Source: PLoS One. 2020 Jul 1;15(7):e0234845. doi: 10.1371/journal.pone.0234845 (PMC7329061; doi:10.1371/journal.pone.0234845)
Supplement: S9 Table — (DOCX) [file pone.0234845.s009.docx]

**Table S9**. Baseline characteristics PD versus no PD validated by GP for Combined cohort.

|  | PD cases | No PD cases | p-value |
| --- | --- | --- | --- |
| *Number of participants (%)* | 85 | 416 |  |
| *Age at baseline* | | | |
| Mean (SD) | 59.52(6.99) | 53.81(9.13) | <0.001 |
| *Sex (%)* | | | |
| Male | 22(25.9%) | 116(27.9%) | 0.808 |
| Female | 63(74.1%) | 300(72.1%) |  |
| *Education (%)* | | | |
| Low | 53(63.1%) | 240(58.5%) | 0.233 |
| Medium | 10(11.9%) | 81(19.8%) |  |
| High | 21(25.0%) | 89(21.7%) |  |
| Missing | 1 | 6 |  |
| *Smoking status at baseline (%)* | | | |
| Never smoker | 49(57.6%) | 165(40.0%) | 0.003 |
| Past smoker | 29(34.1%) | 159(38.6%) |  |
| Current smoker | 7(8.2%) | 88(21.4%) |  |
| Missing | 0 | 4 |  |
| Family history PD 1^st^ degree (%)* | | | |
| Yes | 5(8.6%) | 24 (6.0%) | 0.629 |

*Only available for follow-up 3 in EPIC-NL, % calculated based on these participants.
PD, Parkinson’s Disease; SD, standard deviation; GP, general practitioner.
