# Supplementary material for: Parkinson’s disease case ascertainment in prospective cohort studies through combining multiple health information resources
Source: PLoS One. 2020 Jul 1;15(7):e0234845. doi: 10.1371/journal.pone.0234845 (PMC7329061; doi:10.1371/journal.pone.0234845)
Supplement: S10 Table — (DOCX) [file pone.0234845.s010.docx]

**Table S10.** Agreement different sources of PD information and PD status, among cases validated by the GP in AMIGO, EPIC-NL and Combined cohort.

| Verified Parkinson status AMIGO | | |
| --- | --- | --- |
| Ascertainment source | Parkinson (n=18) | No Parkinson (n=208) |
| Self-reported Parkinson | 15 (83.3%) | 6 (2.9%) |
| Self-reported Parkinson medication | 15 (83.3%) | 11 (5.3%) |
| PD on death certificate | 1 (5.6%) | 0 (0%) |
| EMR registry | 16 (88.9%) | 98 (47.1%) |
| EMR ICPC diagnose | 14 (77.8%) | 3 (1.4%) |
| EMR ATC medication | 14 (77.8%) | 97 (46.6%) |
| Tannerscore 2011 ≥ 5 | 10 (55.6%) | 98 (47.1%) |
| Tannerscore baseline (mean, SD) | 5.00(3.18) | 3.52(2.47) |
| Verified Parkinson status EPIC-NL | | |
|  | Parkinson (n=67) | No Parkinson (n=208) |
| Self-reported Parkinson | 43 (64.2%) | 29 (13.9%) |
| Self-reported Parkinson medication | 35 (52.2%) | 12 (5.8%) |
| PD on death certificate | 9 (13.4%) | 1 (0.5%) |
| HDR registry | 23 (34.3%) | 19 (9.1%) |
| Tannerscore 2011 ≥ 5 | 31 (46.3%) | 85 (40.9%) |
| Tannerscore baseline (mean, SD) | 5.85(2.33) | 3.86(2.07) |
| Verified Parkinson status COMBINED | | |
|  | Parkinson(n=85) | No Parkinson (n=416) |
| Self-reported Parkinson | 58 (68.2%) | 35 (8.4%) |
| Self-reported Parkinson medication | 50 (58.8%) | 23 (5.5%) |
| PD on death certificate | 10 (11.8%) | 1 (0.2%) |
| Tannerscore 2011 ≥ 5 | 41 (48.2%) | 183 (44.0%) |
| Tannerscore baseline (mean, SD) | 5.59(2.62) | 3.69(2.29) |

PD, Parkinson’s Disease; EMR, electronic medical records; HDR, hospital discharge registry; SD, standard deviation.
