# Supplementary material for: Parkinson’s disease case ascertainment in prospective cohort studies through combining multiple health information resources
Source: PLoS One. 2020 Jul 1;15(7):e0234845. doi: 10.1371/journal.pone.0234845 (PMC7329061; doi:10.1371/journal.pone.0234845)
Supplement: S11 Table — (DOCX) [file pone.0234845.s011.docx]

**Table S11.** Baseline characteristics likelihood 3 versus likelihood 0-2 in the Combined cohort.

|  | likelihood 0-2 | likelihood 3 | p-value |
| --- | --- | --- | --- |
| *Number of participants (%)* | 54479 | 346 |  |
| *Age at baseline* | | | |
| Mean (SD) | 49.55(11.29) | 56.71(8.14) | <0.001 |
| *Sex (%)* | | | |
| Male | 16720(30.7%) | 98(28.3%) | 0.372 |
| Female | 37759(69.3%) | 248(71.7%) |  |
| *Education (%)* | | | |
| Low | 28533(52.7%) | 198(57.6%) | 0.194 |
| Medium | 11966(22.1%) | 67(19.5%) |  |
| High | 13662(25.2%) | 79(23.0%) |  |
| Missing | 318 | 2 |  |
| *Smoking status at baseline (%)* | | | |
| Never smoker | 21828(40.2%) | 147(42.9%) | 0.002 |
| Past smoker | 18046(33.2%) | 133(38.8%) |  |
| Current smoker | 14421(26.6%) | 63(18.4%) |  |
| Missing | 184 | 3 |  |
| Family history PD 1^st^ degree (%)* | | | |
| Yes | 1111(3.9%) | 22 (8.9%) | <0.001 |

*Only available for follow-up 3 in EPIC-NL, % calculated based on these participants.
PD, Parkinson’s Disease; SD, standard deviation.
