# Supplementary material for: Parkinson’s disease case ascertainment in prospective cohort studies through combining multiple health information resources
Source: PLoS One. 2020 Jul 1;15(7):e0234845. doi: 10.1371/journal.pone.0234845 (PMC7329061; doi:10.1371/journal.pone.0234845)
Supplement: S12 Table — (DOCX) [file pone.0234845.s012.docx]

**Table S12**. Baseline characteristics of likelihood 3 versus likelihood 0-2 for EPIC-NL and AMIGO.

|  | EPIC-NL | | | AMIGO | | |
| --- | --- | --- | --- | --- | --- | --- |
|  | Likelihood 0-2 | Likelihood 3 | p-value | Likelihood 0-2 | Likelihood 3 | p-value |
| *Number of participants (%)* | 39835 | 176 |  | 14659 | 170 |  |
| *Age at baseline* | | | | | | |
| Mean (SD) | 49.16 (11.90) | 58.82 (7.48) | <0.001 | 50.61(9.37) | 54.52(8.23) | <0.001 |
| *Sex (%)* | | | | | | |
| Male | 10228(25.7%) | 32(18.2%) | 0.029 | 6495 (44.3%) | 66(38.8%) | 0.176 |
| Female | 29607(74.3%) | 144(81.8%) |  | 8164(55.7%) | 104(61.2%) |  |
| *Education (%)* | | | | | | |
| Low | 24075(60.9%) | 123(70.7%) | 0.029 | 4462(30.5%) | 75(44.1%) | 0.001 |
| Medium | 7384(18.7%) | 23(13.2%) |  | 4583(31.3%) | 44(25.9%) |  |
| High | 8067(20.4%) | 28(16.1%) |  | 5605(38.2%) | 51(30.0%) |  |
| Missing | 309 | 2 |  | 9 | 0 |  |
| *Smoking status at baseline (%)* | | | | | | |
| Never smoker | 15157(38.2%) | 86(49.4%) | <0.001 | 6679(45.6%) | 61(36.1%) | 0.013 |
| Past smoker | 12377(31.2%) | 63(36.2%) |  | 5674(38.8%) | 70(41.4%) |  |
| Current smoker | 12139(30.6%) | 25(14.4%) |  | 2284(15.6%) | 38(22.5%) |  |
| Missing | 162 | 2 |  | 22 | 1 |  |
| Family history PD 1^st^ degree (%)* | | | | | | |
| Yes | 628(4.5%) | 10(12.8%) | 0.001 | 484(3.3%) | 12(7.1%) | 0.013 |

*Only available for follow-up 3 in EPIC-NL, % calculated based on these participants.
PD, Parkinson’s Disease; SD, standard deviation.
