# Supplementary material for: Parkinson’s disease case ascertainment in prospective cohort studies through combining multiple health information resources
Source: PLoS One. 2020 Jul 1;15(7):e0234845. doi: 10.1371/journal.pone.0234845 (PMC7329061; doi:10.1371/journal.pone.0234845)
Supplement: S13 Table — (DOCX) [file pone.0234845.s013.docx]

**Table S13.** Crude and adjusted logistic regression analysis of likelihood 3 compared to likelihood 0-2 for the risk factors smoking (baseline), 1^st^ degree family history of PD, and sex in AMIGO, EPIC-NL and Combined cohort.

| AMIGO | | |  |
| --- | --- | --- | --- |
|  | Odds Ratio  [95% CI]; crude* | Odds Ratio  [95% CI]; adjusted** | Odds Ratio  [95% CI]; adjusted*** |
| *Smoking at baseline* | | |  |
| Never smokers | 1.0[Ref] | 1.0[Ref] | 1.0[Ref] |
| Past smokers | 1.35 [0.96-1.91] | 1.10[0.77-1.56] | 1.12[0.79-1.59] |
| Current smokers | 1.82[1.20-2.73] | 1.69[1.11-2.54] | 1.80[1.18-2.69] |
| *1^st^ degree family history of PD* | | |  |
| No first degree family history PD | 1.0[Ref] | 1.0[Ref] | 1.0[Ref] |
| First degree family history of PD | 2.22[1.16-3.86] | 1.90[0.99-3.31] | 1.92[1.00-3.34] |
| *Sex* | | |  |
| Female | 1.0[Ref] | 1.0[Ref] | 1.0[Ref] |
| Male | 0.81[0.59-1.10] | 0.79[0.57-1.07] | 0.76[0.55-1.03] |
| *Age* | | | |
| Age (continuous) | 1.05[1.03-1.07] | 1.05[1.03-1.07] | 1.05[1.03-1.07] |
| EPIC-NL | | |  |
| *Smoking at baseline* | | |  |
| Never smokers | 1.0[Ref] | 1.0[Ref] | 1.0[Ref] |
| Past smokers | 0.90[0.65-1.25] | 0.87 [0.62-1.21] | 0.87[0.62-1.21] |
| Current smokers | 0.37[0.23-0.56] | 0.49[0.30-0.76] | 0.49[0.30-0.76] |
| *1^st^ degree family history of PD* | | |  |
| No first degree family history PD | 1.0[Ref] | 1.0[Ref] | 1.0[Ref] |
| First degree family history of PD | 3.10[1.49-5.77] | 3.09[1.49-5.77] | 3.10[1.49-5.79] |
| *Sex (EPIC-MORGEN only)* | | |  |
| Female | 1.0[Ref] | 1.0[Ref] | 1.0[Ref] |
| Male | 1.65[0.95-2.89] | 1.56[0.90-2.76] | 1.55[0.90-2.72] |
| *Age* | | | |
| Age (continuous) | 1.11[1.09-1.13] | 1.11[1.09-1.14] | 1.11[1.09-1.14] |
| COMBINED |  |  |  |
| *Smoking at baseline* | | |  |
| Never smokers | 1.0[Ref] | 1.0[Ref] | 1.0[Ref] |
| Past smokers | 1.09[0.86-1.38] | 0.94[0.74-1.19] | 0.94[0.74-1.19] |
| Current smokers | 0.77[0.57-1.03] | 0.88[0.65-1.18] | 0.89[0.66-1.20] |
| *1^st^ degree family history of PD* | | |  |
| No first degree family history PD | 1.0[Ref] | 1.0[Ref] | 1.0[Ref] |
| First degree family history of PD | 2.55[1.59-3.88] | 2.29[1.43-3.49] | 2.29[1.43-3.49] |
| *Sex* | | |  |
| Female | 1.0[Ref] | 1.0[Ref] | 1.0[Ref] |
| Male | 0.72[0.56-0.91] | 0.86[0.67-1.11] | 0.85[0.66-1.08] |
| *Age* | | | |
| Age (continuous) | 1.08[1.06-1.09] | 1.08[1.06-1.09] | 1.08[1.06-1.09] |

Likelihood 3 compared to likelihood 0-2. *Adjusted for cohort (only for combined dataset). **Adjusted for age at baseline, baseline education level, sex, cohort(only for combined dataset). *** Adjusted for age at baseline, sex, cohort (only for combined dataset). Ref, reference; PD, Parkinson Disease; CI, Confidence Interval.
