# Supplementary material for: Parkinson’s disease case ascertainment in prospective cohort studies through combining multiple health information resources
Source: PLoS One. 2020 Jul 1;15(7):e0234845. doi: 10.1371/journal.pone.0234845 (PMC7329061; doi:10.1371/journal.pone.0234845)
Supplement: S14 Table — (DOCX) [file pone.0234845.s014.docx]

**Table S14.** Crude and adjusted logistic regression analysis of likelihood 3 compared to likelihood 0-1 and likelihood 3 compared to likelihood 0 for the risk factors smoking (baseline), 1^st^ degree family history of PD, and sex in AMIGO, EPIC-NL and Combined cohort.

| AMIGO | | | | |
| --- | --- | --- | --- | --- |
|  | Likelihood 0-1 | | Likelihood 0 | |
|  | Odds Ratio  [95% CI]; crude* | Odds Ratio  [95% CI]; adjusted** | Odds Ratio  [95% CI]; crude* | Odds Ratio  [95% CI]; adjusted** |
| *Smoking at baseline* | | | | |
| Never smokers | 1.0[Ref] | 1.0[Ref] | 1.0[Ref] | 1.0[Ref] |
| Past smokers | 1.36[0.96-1.92] | 1.10[0.78-1.57] | 1.42[1.00-2.01] | 1.13[0.80-1.62] |
| Current smokers | 1.85[1.22-2.77] | 1.71[1.12-2.57] | 2.02[1.33-3.02] | 1.84[1.21-2.77] |
| *1^st^ degree family history of PD* | | | | |
| No first degree family history PD | 1.0[Ref] | 1.0[Ref] | 1.0[Ref] | 1.0[Ref] |
| First degree family history of PD | 2.25[1.18-3.90] | 1.92[1.00-3.33] | 2.31[1.21-4.01] | 1.95[1.02-3.41] |
| *Sex* | | | | |
| Female | 1.0[Ref] | 1.0[Ref] | 1.0[Ref] | 1.0[Ref] |
| Male | 0.81[0.59-1.10] | 0.79[0.58-1.08] | 0.81[0.59-1.10] | 0.79[0.58-1.08] |
| EPIC-NL | | | | |
| *Smoking at baseline* | | | | |
| Never smokers | 1.0[Ref] | 1.0[Ref] | 1.0[Ref] | 1.0[Ref] |
| Past smokers | 0.91[0.65-1.25] | 0.87[0.62-1.21] | 0.91[0.66-1.26] | 0.88[0.63-1.22] |
| Current smokers | 0.37[0.23-0.56] | 0.49[0.30-0.76] | 0.36[0.23-0.55] | 0.48[0.30-0.74] |
| *1^st^ degree family history of PD* | | | | |
| No first degree family history PD | 1.0[Ref] | 1.0[Ref] | 1.0[Ref] | 1.0[Ref] |
| First degree family history of PD | 3.10[1.49-5.78] | 3.10[1.49-5.79] | 3.15[1.51-5.87] | 3.16[1.51-5.93] |
| *Sex (EPIC-MORGEN only)* | | | | |
| Female | 1.0[Ref] | 1.0[Ref] | 1.0[Ref] | 1.0[Ref] |
| Male | 1.64[0.95-2.88] | 1.55[0.89-2.75] | 1.63[0.94-2.85] | 1.54[0.88-2.72] |
| COMBINED | | | | |
| *Smoking at baseline* | | | | |
| Never smokers | 1.0[Ref] | 1.0[Ref] | 1.0[Ref] | 1.0[Ref] |
| Past smokers | 1.09[0.86-1.38] | 0.94[0.74-1.20] | 1.12[0.89-1.42] | 0.96[0.76-1.22] |
| Current smokers | 0.77[0.57-1.04] | 0.88[0.65-1.18] | 0.79[0.58-1.06] | 0.89[0.66-1.20] |
| *1^st^ degree family history of PD* | | | | |
| No first degree family history PD | 1.0[Ref] | 1.0[Ref] | 1.0[Ref] | 1.0[Ref] |
| First degree family history of PD | 2.57[1.60-3.91] | 2.30[1.43-3.50] | 2.63[1.64-4.00] | 2.32[1.45-3.55] |
| *Sex* | | | | |
| Female | 1.0[Ref] | 1.0[Ref] | 1.0[Ref] | 1.0[Ref] |
| Male | 0.72[0.56-0.91] | 0.86[0.67-1.11] | 0.70[0.55-0.89] | 0.85[0.66-1.09] |

Likelihood 3 compared to likelihood 0-1 and likelihood 0. *Adjusted for cohort(only for combined dataset). **Adjusted for age at baseline, baseline education level, sex, cohort. Ref, reference; PD, Parkinson Disease; CI, Confidence Interval.
