# Supplementary material for: Parkinson’s disease case ascertainment in prospective cohort studies through combining multiple health information resources
Source: PLoS One. 2020 Jul 1;15(7):e0234845. doi: 10.1371/journal.pone.0234845 (PMC7329061; doi:10.1371/journal.pone.0234845)
Supplement: S15 Table — (DOCX) [file pone.0234845.s015.docx]

**Table S15.** Crude and adjusted logistic regression analysis of confirmed cases by GP for the risk factors smoking (baseline), 1^st^ degree family history of PD, and sex in Combined cohort.

| COMBINED | | |
| --- | --- | --- |
|  | Odds Ratio  [95% CI]; crude* | Odds Ratio  [95% CI]; adjusted** |
| *Smoking at baseline* | | |
| Never smokers | 1.0[Ref] | 1.0[Ref] |
| Past smokers | 0.70[0.44-1.11] | 0.63[0.39-1.00] |
| Current smokers | 0.20[0.08-0.42] | 0.27[0.11-0.56] |
| *1^st^ degree family history of PD* | | |
| No first degree family history PD | 1.0[Ref] | 1.0[Ref] |
| First degree family history of PD | 2.27[0.79-5.19] | 2.11[0.73-4.84] |
| *Sex* | | |
| Female | 1.0[Ref] | 1.0[Ref] |
| Male | 0.75[0.44-1.21] | 1.34[0.76-2.29] |

*Adjusted for cohort. **Adjusted for age at baseline, baseline education level, gender, cohort. Ref, reference; PD, Parkinson Disease; CI, Confidence Interval; GP general practitioner Reference group= likelihood 0.
