# Supplementary material for: Parkinson’s disease case ascertainment in prospective cohort studies through combining multiple health information resources
Source: PLoS One. 2020 Jul 1;15(7):e0234845. doi: 10.1371/journal.pone.0234845 (PMC7329061; doi:10.1371/journal.pone.0234845)
Supplement: S16 Table — (DOCX) [file pone.0234845.s016.docx]

|  | Odds Ratio  [95% CI]; EMR with medication | Odds Ratio  [95% CI]; EMR without medication |
| --- | --- | --- |
| *Smoking at baseline* | | |
| Never smokers | 1.0[Ref] | 1.0[Ref] |
| Past smokers | 1.35 [0.96-1.91] | 1.27[0.73-2.23] |
| Current smokers | 1.82[1.20-2.73] | 2.06[1.09-3.83] |
| *1^st^ degree family history of PD* | | |
| No first degree family history PD | 1.0[Ref] | 1.0[Ref] |
| First degree family history of PD | 2.22[1.16-3.86] | 2.34[0.82-5.30] |
| *Sex* | | |
| Female | 1.0[Ref] | 1.0[Ref] |
| Male | 0.81[0.59-1.10] | 1.65[1.02-2.71] |

**Table S16**. Likelihood 3 with and without medication diagnosis in the electronic medical registry compared to likelihood 0-2 for the risk factors smoking (baseline), 1^st^ degree family history of PD, and sex within AMIGO.

^*^ Controls were all participants with likelihood score 0-2.
LH, likelihood; Ref, reference; PD, Parkinson Disease; CI, Confidence Interval.
