# Supplementary material for: Parkinson’s disease case ascertainment in prospective cohort studies through combining multiple health information resources
Source: PLoS One. 2020 Jul 1;15(7):e0234845. doi: 10.1371/journal.pone.0234845 (PMC7329061; doi:10.1371/journal.pone.0234845)
Supplement: S17 Table — (DOCX) [file pone.0234845.s017.docx]

**Table S17.** Logistic regression analysis of likelihood 3 compared to likelihood 0 for the risk factors smoking (baseline), 1^st^ degree family history of PD in AMIGO, EPIC-NL and Combined cohort stratified by sex and age.

| AMIGO | | | | |
| --- | --- | --- | --- | --- |
|  | Odds Ratio  [95% CI]; female | Odds Ratio  [95% CI]; male | Odds Ratio  [95% CI]; < 60 years | Odds Ratio  [95% CI]; >=60 years |
| *Smoking at baseline* | | | | |
| Never smokers | 1.0[Ref] | 1.0[Ref] | 1.0[Ref] | 1.0[Ref] |
| Past smokers | 1.43 [0.92-2.23] | 1.40[0.80-2.47] | 1.46[0.93-2.30] | 1.00[0.58-1.74] |
| Current smokers | 1.93[1.12-3.25] | 2.17[1.12-4.10] | 2.55[1.57-4.13] | 1.13[0.47-2.45] |
| *1^st^ degree family history of PD* | | | | |
| No first degree family history PD | 1.0[Ref] | 1.0[Ref] | 1.0[Ref] | 1.0[Ref] |
| First degree family history of PD | 3.09[1.44-5.89] | 1.33[0.32-3.63] | 2.82[1.25-5.50] | 1.37[0.41-3.40] |
| EPIC-NL | | | | |
| *Smoking at baseline* | | | | |
| Never smokers | 1.0[Ref] | 1.0[Ref] | 1.0[Ref] | 1.0[Ref] |
| Past smokers | 0.91[0.63-1.31] | 1.01[0.46-2.20] | 0.71[0.44-1.12] | 1.30[0.81-2.08] |
| Current smokers | 0.42[0.25-0.67] | 0.24[0.07-0.69] | 0.35[0.19-0.61] | 0.63[0.28-1.26] |
| *1^st^ degree family history of PD* | | | | |
| No first degree family history PD | 1.0[Ref] | 1.0[Ref] | 1.0[Ref] | 1.0[Ref] |
| First degree family history of PD | 3.21[1.47-6.21] | 2.39[0.13-12.49] | 2.53[0.97-5.48] | 5.41[1.52-15.25] |
| COMBINED | | | | |
| *Smoking at baseline* | | | | |
| Never smokers | 1.0[Ref] | 1.0[Ref] | 1.0[Ref] | 1.0[Ref] |
| Past smokers | 1.09[0.83-1.44] | 1.27[0.81-2.00] | 1.04[0.75-1.42] | 1.14 [0.80-1.64] |
| Current smokers | 0.75[0.52-1.06] | 1.02[0.57-1.78] | 0.95[0.66-1.37] | 0.79[0.45-1.33] |
| *1^st^ degree family history of PD* | | | | |
| No first degree family history PD | 1.0[Ref] | 1.0[Ref] | 1.0[Ref] | 1.0[Ref] |
| First degree family history of PD | 2.92[1.72-4.67] | 1.48[0.45-3.60] | 2.48[1.37-4.16] | 2.23[0.98-4.42] |

^*^ Controls were all participants with likelihood score 0.
LH, likelihood; Ref, reference; PD, Parkinson Disease; CI, Confidence Interval.
